# Economic impact of heart failure in Brazil

**DOI:** 10.7189/jogh.16.04082

**Published:** 2026-05-15

**Authors:** Nathalia Volpi e Silva, Daniela Vicentini, Renato Lima Vitorasso, Vicky Nogueira-Pileggi, Elizabeth Bilevicius, Vivian Cardoso Batista, Renato Mantelli Picoli

**Affiliations:** 1 Researcher, Development and Medical Affairs, Viatris, São Paulo, Brazil; 2 Oracle Life Sciences, São Paulo, Brazil

## Abstract

**Background:**

Heart failure (HF) is highly prevalent worldwide and is the main cause of hospitalisations in developed countries. It is the leading cause of hospitalisations for cardiovascular diseases in Brazil, which places a substantial burden on the Unified Health System. We aimed to evaluate public healthcare expenditures for patients diagnosed with HF and those potentially at risk of developing the condition.

**Methods:**

We used data from the *DATASUS* database, which encompasses information from the Brazilian Unified Health System (*Sistema Único de Saúde* (*SUS*)). We utilised the Outcome Information System Ambulatory – *SUS* (*Sistema de Informações Hospitalares do SUS* (*SIA-SUS*)) and the Hospital Information System – SUS (*Sistema de Informações Hospitalares do SUS* (*SIH-SUS*)) systems. After evaluating public health expenditures for patients with or at risk of developing HF, we analysed the distribution of these expenditures according to demographic factors and clinical characteristics to determine the financial impact on the healthcare system and its relationship with different patient profiles.

**Results:**

The *SIA-SUS* comprised 354 171 patients diagnosed with HF, while the *SIH-SUS* contributed 708 161 patients as a potential HF group. We linked 67 539 patients from *SIA-SUS* and *SIH-SUS* due to the need for a proxy identifier. Overall, potential HF patients incurred higher costs than diagnosed HF patients, with this disparity being particularly evident in hospital-related expenses. From 2018 to 2019, the average costs for potential HF patients were 1.7 times greater than those for diagnosed HF patients. Potential HF patients incurred the highest cost per patient across all age groups, with those ≤44 years old incurring the highest costs (USD 501.04), followed by those 45–64 years old (USD 488.76). Group diagnosed with HF, patients aged 45–64 years incurred the highest costs (USD 419.89), followed by those aged ≥65 (USD 330.02). Males incurred the highest costs in both diagnosed and potential HF groups.

**Conclusions:**

The burden of potential HF was higher than diagnosed HF, showed by greater public healthcare expenditures. Understanding the cost of this disease is important for informing resource allocation and the implementation of more effective measures for the population with HF.

Heart failure (HF) is highly prevalent worldwide [1] and is the main cause of hospitalisations in developed countries. It is the leading cause of hospitalisations for cardiovascular diseases in Brazil [2], the third leading cause of hospitalisations in general [3], and is associated with increasing healthcare resources utilisation and rising costs [4]. These high hospitalisation and mortality rates were responsible for health services expenses that exceeded Brazilian Reais (BRL) 3 billion (USD 626.5 million) in 2019 [5]. Additionally, in 2018, the economic burden of HF ranked as the second-highest financial cost (BRL 22.1 billion/USD 6.8 billion) [6].

In Brazil, HF places a substantial burden on the Unified Health System (*Sistema Único de Saúde* (*SUS*)), as its management is highly resource-intensive, with patients often requiring repeated hospital admissions, long-term pharmacological treatment, and frequent outpatient monitoring. Despite the high burden, there is a lack of comprehensive data regarding the financial costs associated with the diagnosis and treatment of HF, particularly in relation to the expenditures on both ambulatory and hospital services.

The *SUS* is Brazil’s publicly funded and universal healthcare system, recognised as the largest of its kind in the world, providing free, comprehensive, and equitable health services to all Brazilians. According to the Brazilian Ministry of Health, approximately 70% of the Brazilian population relies exclusively on the *SUS* for healthcare [7]. *DATASUS* is the information technology department of Brazil’s Ministry of Health, responsible for collecting, managing, and disseminating health data from the SUS, and serving as the main source for public health statistics and research in the country. A previous *DATASUS*-based study in Brazil found that each year, approximately 54 000 patients per year get misdiagnosed, and 200 000 deaths could be attributed to HF. Literature further indicates that most HF cases present acutely, with abrupt symptoms that hinder timely diagnosis and can lead to death [8]. Such misdiagnoses can result in incorrect or ineffective treatment, creating unnecessary costs and complications in the short term, as well as increased healthcare expenses in the long run. These findings highlight the importance of accurate diagnosis and a comprehensive approach to identifying potential cases of HF to improve the recording and management of this condition.

In light of these considerations, our research question was: ‛What is the magnitude and distribution of public healthcare expenditures associated with patients diagnosed with HF and those potentially at risk of the condition in Brazil?’ We hypothesised that patients potentially at risk of HF incur higher healthcare costs compared to those with a confirmed diagnosis, due to more frequent hospital admissions, complex clinical needs, and the consequences of suboptimal initial management.

We aimed to evaluate public healthcare expenditures in both ambulatory and hospital systems for patients diagnosed with HF and those at risk of developing the condition. Additionally, we wanted to analyse the distribution of these expenditures according to demographic factors (geographic region, sex, age) and clinical characteristics (types of procedures performed), providing a comprehensive view of the financial impact of HF on the healthcare system and its relationship with different patient profiles.

## METHODS

### Data extraction

We obtained all data from the *DATASUS* database, which provides information from the SUS. We utilised the Outcome Information System Ambulatory (*Sistema de Informações Hospitalares do SUS* (*SIA-SUS*)) and Hospital Information System (*Sistema de Informações Hospitalares do SUS* (*SIH-SUS*)) systems. We retrieved all files directly from the *DATASUS* repository via a file transfer protocol [9]. We converted the data sets, originally available in .dbc format, into the .parquet format for processing and analysis. We conducted data extraction and variable calculations using *R*, version 4.4.1 (R Core Team, Vienna, Austria) and Python, version 3.12.5 (Python Software Foundation, Wilmington, Delaware, USA). For the descriptive and statistical analyses, we utilised the ‘pandas’ and ‘stats models’ libraries in Python.

### Inclusion criteria

**Figure 1:**
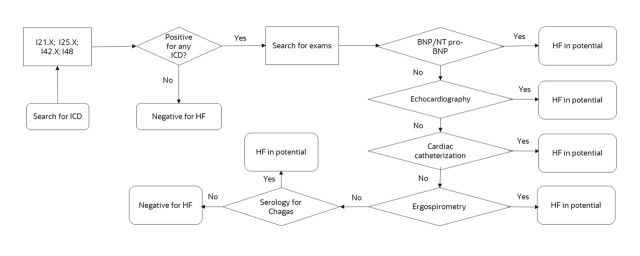
Flowchart for identifying sub notified patients by ICD and Procedure in *SIA-SUS*.

To identify target patients, we used the selection criteria previously described by Batista et al [8]. This approach focuses on two groups: patients diagnosed with HF based on International Classification of Diseases, 10th Revision (ICD-10) code I50, and those classified as potential HF (**Figure 1**).

We set the study period from 1 January 2018 to 31 December 2022. However, we also considered data from the preceding three years (1 January 2015) to account for patients who may have had HF or potential HF records before 2018. This approach enabled us to identify patients diagnosed with ICD-10 code I50 specifically during the study period, minimising bias in the cases classification in the initial years of the analysis.

We identified diagnosed HF patients based on the presence of at least one record of ICD-10 I50 in either the ambulatory procedure authorisation (APAC) from the *SIA-SUS* or the *SIH-SUS* record system. The potential HP group consisted of patients without any ICD-10 I50 records, whom we also identified in the *SIA-SUS* system (**Figure 1**).

As there is no unique identifier linking *SIA-SUS* and *SIH-SUS* records in the *DATASUS* database, we constructed a proxy ID by concatenating ZIP codes, sex, and dates of birth to probabilistically link records across systems. To improve accuracy, we first performed a data harmonisation within the *SIA-SUS* data set, which contains a unique patient identifier. For each patient, we calculated the mode of ZIP code, sex, and date of birth across all their ambulatory records to define a consistent set of demographic variables. We then used these standardised values to create the proxy ID, which was subsequently matched against *SIH-SUS* records. Although this deterministic approach carries a risk of mislinking or misclassification, a similar linkage strategy has been successfully applied in the integration of Brazilian health system data, achieving sensitivity and specificity above 95% [10]. Nonetheless, such a proxy may still introduce non-differential misclassification bias, particularly in densely populated regions.

To assess the robustness of our linkage, we performed internal consistency checks of clinical trajectories across ambulatory and hospital data, including temporal coherence between diagnoses and procedures. Despite these safeguards, residual linkage errors remained a limitation inherent to administrative data sets that lack a unique patient identifier.

The extracted variables included demographic information (sex and age group), procedures performed, ICD codes associated with procedures, and cost values. We obtained cost data directly from the *SIA-SUS* and *SIH-SUS* data sets, reflecting the amounts reimbursed by the Brazilian government for each APAC and for hospital admissions. We conducted data cleaning, harmonisation, filtering, and processing to ensure transparency, consistency, and reproducibility of our findings.

### Descriptive and statistical analysis

We excluded outliers deviating by more than 0.0008% from the sample in both systems and groups to estimate the mean annual cost per patient. Although heart transplants are common among HF patients, none of the excluded individuals had undergone this procedure.

We converted cost data from the *DATASUS* database, initially recorded in BRL, to USD using the monthly average exchange rate provided by the Central Bank of Brazil, covering the period from 8 July to 5 August 2024. The exchange rate applied was BRL 5.53 per 1 USD.

We calculated descriptive statistics, including counts, means, and standard deviations (SDs) for all analysed variables. To compare health expenditures between the ICD-10 I50 group and the potential HF group, we fitted generalised linear mixed-effects models, both unadjusted and adjusted for sex, region, and age. The models used the Tweedie distribution with a log link, which is appropriate for nonnegative, right-skewed outcomes that may include zeros.

All models included a random intercept at the patient level (Patient ID) to account for within-cluster correlation. We reported the results as exponentiated coefficients, interpreted as mean ratios with their respective 95% confidence intervals (CIs), based on Wald standard errors. We set statistical significance at a two-sided *P*-value <0.05.

We specified a crude univariable model as value dollar ~ group + (1 | patient ID), estimating the association between the exposure variable (group) and mean group expenditure. We then specified an adjusted multivariable model as value dollar ~ group + year + gender + age group + (1 | patient ID), adjusting for calendar year (year), sex (gender), and modal age group (age group).

All analyses were performed in R (version X.Y.Z) using base functions and the *R* packages ‘dplyr’, ‘ggplot2’, and ‘generalized linear mixed-effects models TMB’. A specific random seed was set to ensure reproducibility of results.

## RESULTS

We retrieved data on 354 171 HD-diagnosed patients in the *SIA-SUS* and 708 161 in the *SIH-SUS*. From these, we identified 399 133 patients in the *SIA-SUS* as potential HF group. A linkage between patients from *SIA-SUS* and patients from *SIH-SUS*, matched 67 539 due to the need for a proxy identifier. As the proxy identification relies solely on ambulatory criteria, the extrapolation of potential HF cases to the SIH was compromised, hindering us from accurately assessing the total annual expenditure for these patients, as the data would not adequately reflect the reality. Given this, we calculated the average annual cost per patient instead; both samples were sufficiently large to allow for generalisation, thereby better representing the *DATASUS* reality.

We initially had 354 174 HF patients in the *SIA-SUS* and 708 168 in the *SIH-SUS*; we retained 354 171 and 708 161, respectively, after removing outliers. Similarly, we had 399 137 potential HF cases in the SIA-SU and 67 539 in the *SIH-SUS*, and we retained 399 133 and 67 539, respectively, after removing outliers.

After adjusting for year, sex, age group, and region, potential HF patients incurred significantly higher mean costs compared to those with a confirmed HF diagnosis. In the ambulatory setting (**Table 1**), potential HF patients had an average of 1.91 times higher expenditures than those with an HF diagnosis (exp(*β*) = 1.91; 95% CI = 1.90–1.92, *P* < 0.001). Similarly, in the hospital system (**Table 1**), the regression model estimated that the potential HF group incurred 1.71 times higher mean costs (exp(*β*) = 1.71; *P* < 0.001). We also observed regional and temporal effects, with lower costs in the North, Southeast, and South, and typical year-on-year variations.

**Table 1:** Determinants of healthcare expenditures in hospital and ambulatory care: results from adjusted GLMM with Tweedie distribution among diagnosed and potential heart failure patients

Variable	Ambulatorial system		Hospital system	
**Estimate (exp(*β*)) (95% CI)**	***P*-value**	**Estimate (exp(*β*)) (95% CI)**	***P*-value**
Group				
*Potential*	1.91 (1.90, 1.92)	<0.001	1.71(1.00,5.53)	<0.001
Year				
*2019*	0.95 (0.94, 0.95)	<0.001		
*2020*	0.87 (0.87, 0.88)	<0.001		
*2021*	0.84 (0.84, 0.85)	<0.001		
*2022*	0.93 (0.92, 0.93)	<0.001	1.08 (1.00,1.08)	<0.001
Sex				
*Male*	1.17 (1.17, 1.18)	<0.001	1.08 (1.00,1.08)	<0.001
Age in years				
*≥65*	1.19 (1.18, 1.20)	<0.001	0.75 (0.75, 1.00)	<0.001
*45–64*	1.25 (1.24, 1.26)	<0.001	0.88 (0.88, 1.00)	<0.001
Region				
*Northeast*	1.16 (1.14, 1.17)	<0.001	0.85 (0.85, 1.00)	<0.001
*North*	0.67 (0.66, 0.68)	<0.001	0.79 (0.79, 1.01)	<0.001
*Southeast*	0.75 (0.74, 0.76)	<0.001	0.98 (0.98, 1.00)	<0.001
*South*	0.44 (0.43, 0.44)	<0.001	1.02 (1.00, 1.02)	<0.001

CI – confidence interval, exp(*β*) – exponentiated coefficient

These findings remained consistent in the descriptive annual per-patient cost data (**Table 2**). Throughout the analysed period (2018–2022), potential HF patients consistently incurred higher mean costs in both hospital and ambulatory care compared to confirmed HF patients. For example, in the hospital system, average costs per potential HF patient increased from a mean of USD 1082.14 (SD = 1495.25) in 2018 to USD 1312.14 (SD = 1763.13) in 2022, while diagnosed HF patients saw a rise from a mean of USD 634.94 (SD = 1413.29) to USD 872.62 (SD = 1759.71) over the same period.

**Table 2:** Cost per patient in ambulatorial and hospital system of diagnosed and potential HF patients*

		Ambulatorial system	Hospital system	
**Year**	**Group**	**Cost per patient in USD**		**Cost per patient in USD**
2018	HF	105.19 (532.03)		634.94 (1413.29)
	Potentials HF	156.31 (676.13)		1082.14 (1495.25)
2019	HF	113.63 (551.01)		670.91 (1470.39)
	Potentials HF	161.44 (687.53)		1116.30 (1584.00)
2020	HF	119.92 (605.32)		747.49 (1575.84)
	Potentials HF	163.62 (724.39)		1139.64 (1548.70)
2021	HF	130.61 (641.89)		823.39 (1734.09)
	Potentials HF	164.42 (703.18)		1228.89 (1779.33)
2022	HF	160.72 (688.60)		872.62 (1759.71)
	Potentials HF	180.50 (789.17)		1312.14 (1763.13)
Total	HF	403.83 (2003.32)		1135.69 (2140.56)
	Potentials HF	502.92 (2364.43)		1782.57 (2171.81)

HF – heart failure, SD – standard deviation, x̄ – mean

*Values are presented as x̄ (SD).

Clinical and surgical procedures, particularly surgical interventions (*e.g.* coronary angioplasty with stenting), represent major cost drivers among potential HF patients in the hospital system, while complex diagnostic and clinical management pathways have the same effect in ambulatory care. Medications with the highest per-patient cost, such as Sildenafil in the potential group and Sacubitril/Valsartan in the diagnosed HF group, also reflect distinctive patterns of healthcare use (Figures S1–5 in the **Online Supplementary Document**).

Hospital service costs, professional fees, and intensive care unit costs were consistently higher among potential HF patients each year. Male patients incurred notably higher expenses, especially in the potential HF group, where resource allocation for men was nearly twice that of women. Age-stratified costs showed that the 45–64-year age group accounted for a disproportionately high share of expenditures, representing 34.54% of diagnosed HF costs and 51.06% of potential HF costs – highlighting the wider socioeconomic impact beyond the elderly population (**Table 3**).

**Table 3:** Costs per patient of hospital system by type of services for diagnosed and potential HF patients*

Variable	Group	Hospital services in USD	Professional in USD	ICU in USD
Sex				
*Male*	HF	529.72 (1483.49)	87.70 (303.76)	193.06 (834.60)
	Potential HF	928.21 (1644.33)	213.08 (393.86)	260.11 (826.48)
*Female*	HF	445.24 (1256.56)	72.67 (264.39)	174.22 (794.52)
	Potential HF	526.41 (1285.16)	114.95 (300.45)	158.82 (691.58)
Age in years				
*≤44*	HF	122.28 (959.45)	28.40 (253.61)	48.74 (580.06)
	Potential HF	75.67 (676.10)	16.94 (157.44)	20.48 (278.42)
*45 to 64*	HF	327.21 (1202.48)	55.40 (229.86)	116.55 (640.33)
	Potential HF	713.06 (1483.39)	167.49 (356.79)	190.00 (714.46)
*≥65*	HF	525.46 (1200.64)	76.58 (216.67)	201.98 (764.65)
	Potential HF	665.88 (1337.32)	143.59 (315.60)	208.45 (760.60)
Year				
*2018*	HF	547.09 (1201.64)	87.33 (262.58)	171.16 (691.22)
	Potential HF	884.63 (1261.60)	196.07 (320.66)	212.28 (620.33)
*2019*	HF	578.03 (1254.35)	92.50 (266.70)	184.82 (707.93)
	Potential HF	913.18 (1319.98)	202.50 (337.69)	218.83 (664.39)
*2020*	HF	643.84 (1344.20)	103.57 (282.17)	242.40 (844.34)
	Potential HF	946.87 (1321.29)	194.34 (308.80)	271.11 (801.70)
*2021*	HF	709.61 (1484.81)	113.66 (302.43)	290.79 (1018.68)
	Potential HF	1023.53 (1519.15)	206.19 (333.77)	324.08 (978.02)
*2022*	HF	740.09 (1464.29)	132.48 (355.51)	330.77 (1013.27)
	Potential HF	1030.29 (1416.69)	281.77 (446.37)	358.34 (908.18)

HF – heart failure, ICU – Intensive Care Unit, SD – standard deviation, x̄ – mean

*Values are presented as x̄ (SD).

Regarding regional effects, expenditures were notably lower in the North (exp(*β*) = 0.79), Southeast (exp(*β*) = 0.98), and Northeast (exp(*β*) = 0.85), and marginally higher in the South (exp(*β*) = 1.02), all with *P* < 0.001, when compared to the reference region. Yearly trends indicated that each successive year was associated with an approximate 8% increase in costs (exp(*β*) = 1.08). 

The adjusted generalised linear mixed-effects models using Tweede distribution showed that patients in the potential HF in ambulatorial system group had, on average, 1.91 times higher expenditures compared to those in the ICD-10 I50 diagnosed group (exp(*β*) = 1.91; 95% CI = 1.90–1.92; *P* < 0.001) (**Table 1**). Additionally, male patients and those in older age groups incurred significantly higher mean healthcare costs to the public healthcare system. Mean costs were 17% higher for men than for women (exp(*β*) = 1.17; 95% CI = 1.17–1.18), and 25% (exp(*β*) = 1.25; 95% CI = 1.24–1.26) and 19% (exp(*β*) = 1.19; 95% CI = 1.18–1.20) higher for individuals aged 45–64 and ≥65 years, respectively, compared with younger groups.

We also observed regional differences; expenditures were higher in the Northeast (exp(*β*) = 1.16), while lower costs were seen in the North (exp(*β*) = 0.67), Southeast (exp(*β*) = 0.75), and South (exp(*β*) = 0.44) regions, all with *P* < 0.001. Temporal trends indicate a reduction in costs in subsequent years compared to the reference year, with the lowest observed in 2021 (exp(*β*) = 0.84; 95% CI = 0.84–0.85).

In the ambulatory system, clinical procedures constituted more than 50% of total costs for both the diagnosed and potential HF groups. In the hospital system, clinical procedures remained the largest component in the diagnosed group (61.36% of total hospital costs). However, for the potential group, surgical procedures were the main cost driver, accounting for 67.62% of total hospital expenditure (Table S1 in the **Online Supplementary Document**).

Atorvastatin, 40 mg, was the most commonly used drug in both groups. Sildenafil, 20 mg, had the highest cost for the potential HF group. For the diagnosed HF group, Sacubitril/Valsartan 200 mg had the highest cost, while Sildenafil was ranked fourth (Figure S1 in the **Online Supplementary Document****)**.

For diagnostic procedures, the complete blood count and creatinine dosage were the first and second most frequent procedures in the diagnosed and potential HF groups. The cardiac catheterisation was the seventh most frequent procedure in the potential group and the one with the highest cost per patient for both groups. Myocardial scintigraphy for stress and rest perfusion ranked third and fourth for the diagnosed HF group, and second and third for the potential HF group (Figure S2 in the **Online Supplementary Document**).

As for clinical procedures, medical consultation in specialised care was the most frequent for both groups. Physiotherapeutic attendance for motor alterations ranked second in the potential group and only fifth in the diagnosed group. Consultations with higher-level professionals in specialised care were positioned second in the HF diagnosed group and sixth for the potential HF group. Haemodialysis had the highest cost per patient in both groups, but ranked only fifth in terms of the number of patients in the potential HF group (Figure S3 in the **Online Supplementary Document**).

For diagnosed HF, the most common and the highest cost surgery was treatment with multiple surgeries. As for the potential HF patients, the first and second most common surgeries are coronary angioplasties with implantation of one and two stents, respectively. The surgery with the highest costs for the potential HF patients is the coronary angioplasty with implantation of two stents (Figure S4 in the **Online Supplementary Document**).

For clinical procedures, the treatment for HF is the most common and has the highest cost for diagnosed HF. As for potential HF, the treatment for HF ranks second in cost and count, with the treatment for acute myocardial infarction being the most common and highest in cost for potential HF (Figure S5 in the **Online Supplementary Document**).

We categorised the costs of the hospital system by hospital services, professional fees, and intensive care unit costs, broken down by year, sex, and age group. Across both groups, hospital services consistently account for the highest costs per patient over the five years. A considerable portion of resources was directed towards the 45–64 age group, accounting for 34.54% of costs for the diagnosed group and 51.06% for the potential group (**Table 3**).

We also described the cost per patient by age group and sex for diagnosed and potential HF patients in the ambulatory system. Potential HF patients incurred the highest cost per patient across all age groups, with those aged ≥44 years incurring the highest costs (USD 501.04), followed by those aged 45–64 years (USD 488.76). For the diagnosed HF group, patients aged 45–64 years have the highest costs (USD 419.89), followed by those aged ≥65 years (USD 330.02). Regarding the comparison by sex, males incurred the highest costs per patient for both diagnosed and potential HF groups. The North region exhibited the highest cost per patient for potential HF patients in the hospital system, while it had the lowest costs for diagnosed HF patients **(**Figure S6, Panels A and B in the **Online Supplementary Document**). In this region, potential HF patients incurred 2.3 times more expenses compared to diagnosed HF patients. In contrast, in the ambulatory system, the North region presented relatively similar costs for both groups, with diagnosed HF patients incurring slightly higher expenses than potential HF patients (Figure S7, Panels A and B in the **Online Supplementary Document**).

The Northeast region had the highest ambulatory system costs for both groups compared to other Brazilian states. Here, diagnosed HF patients’ costs are 1.8 times higher than those of potential HF patients. The hospital system showed an opposite trend: diagnosed HF patients incur an average cost of USD 859.03, while potential HF patients have a much higher average cost of USD 1764.54, exceeding that of diagnosed HF patients by over 2-fold.

In the remaining regions, the cost differences between both groups in the ambulatory system were generally similar, except for the South, where diagnosed HF patients spent 1.7 times less than potential HF patients. In the hospital system across these regions, the cost differences between diagnosed and potential HF patients ranged from 1.5 to 1.7 times higher for potential HF patients.

## DISCUSSION

To the best of our knowledge, this is the first economic analysis of HF and its potential misdiagnosed patients using public data in Brazil.

Studies have shown that undiagnosed HF often results in more frequent hospital admissions and longer stays due to delayed treatment, which increases the overall cost burden on healthcare systems [11]. For example, our adjusted generalised linear mixed-effects models analysis demonstrated that, in the hospital system, expenditures for patients in the potential HF group were 71% higher than for diagnosed HF patients (exp(*β*) = 1.71; 95% CI = 1.70–1.72, *P* < 0.001). Similarly, in the ambulatory system, potential HF patients incurred nearly double the expenditures when compared to diagnosed HF (exp(*β*) = 1.91; 95% CI = 1.90–1.92, *P* < 0.001). Additionally, these patients frequently present with advanced disease or complications that could have been managed more efficiently if HF had been identified earlier, potentially avoiding high-cost interventions such as intensive care unit admissions and surgeries.

HF is a major medical and economic issue worldwide [12,13]. Data from the USA show markedly higher hospitalisation costs. Charges billed to insurers or patients can exceed USD 50 000, illustrating the inflationary effect of a market-based system with strong reliance on private insurance and less centralised price regulation. Hospitalisations account for up to 73% of total direct HF costs, a pattern similar to Brazil, but the absolute values differ substantially [14]. An overview published in 2021 showed that the cost varies from less than 1000 USD per patient in low-income countries to between EUR 5000 and EUR 15 000 in Europe, and between USD 17 000 and USD 30 000 in the USA [15]. These differences are partly explained by purchasing power parity and resource use intensity, but also by systemic factors such as national health insurance in South Korea, which negotiates standardised tariffs *vs.* the fragmented multi-payer environment in the USA. In Europe, where social insurance systems dominate, higher costs often reflect more extensive use of diagnostics, pharmacotherapy, and long-term care services [13]. We found that the costs with patients in both ambulatory and hospital settings were indeed less than USD 1000; in contrast, the cost for the healthcare system could increase more than 70% when we look at patients misdiagnosed. A review published in 2018 highlighted that hospitalisations represent the highest costs associated with this disease worldwide [13]. Another review from 2022 showed that the clinical burden of hospitalisation for HF in the USA varies from USD 7094 to USD 9769 (median) and USD 10 737 to USD 17 830 (mean) with hospitalisation costs [14]. Indeed, we noted the same pattern of higher cost per patient hospitalisation in Brazil in our analysis. One possible explanation for these findings is that patients who are not properly diagnosed do not receive appropriate treatment, leading to unnecessary expenses due to ineffective therapy. Additionally, proxies used to select these potential HF cases may inherently capture patients who are more complex or severe, which could contribute to higher costs. Moreover, further complications arising from the absence of an accurate diagnosis can result in additional, and potentially avoidable, future healthcare expenditures.

We could not find any literature that describes the costs of the ambulatory system of HF. A scoping review, however, found evidence that multicomponent interventions can reduce HF hospitalisations, and despite their complexity (which includes ambulatory approaches with interdisciplinary teams and a greater number of participating disciplines) appear to enhance effectiveness [16]. Here, we saw that costs within the ambulatory system are 50% lower than within the hospital system, which could indicate a path to treatment in this population. Interestingly, hospital costs were lower in regions in Brazil where the ambulatory costs were higher (Figures S6 and S7 in the **Online Supplementary Document**), possibly indicating that a proper patient follow-up could reduce general costs.

Regarding costs of surgical and clinical procedures, we noted that treatment for multiple surgeries and treatment of heart failure have the highest cost; among HF patients, while the coronary angioplasty with stent implementation and treatment of acute myocardial infarction have the highest cost for potential cases of HF (Figures S4 and S5 in the **Online Supplementary Document**). A study published in 2018 showed that myocardial infarction is the greatest financial cost in Brazil, followed by HF [17], corroborating our findings.

We note there is a large difference between the costs of potential HF and the patients with diagnosed HF in *DATASUS*. We could not find any studies in the literature that evidence the costs in both populations or in the undiagnosed population that might have HF. This provides insights on how to reduce costs in the public health system in Brazil and could guide actions to improve both costs and health of HF patients.

Patients with potential HF, *i.e.* those at risk for or potentially affected by the condition, can impose a substantial financial burden on healthcare systems, often leading to higher overall costs compared to patients with an established diagnosis of HF. Understanding the economic implications of this diagnostic gap is crucial for informing healthcare policies that promote timely detection and intervention, ultimately leading to more cost-effective management and improved patient quality of life. Early identification and treatment of HF could significantly reduce preventable hospitalisations and reduce the long-term financial impact on health systems.

### Limitations

One of the principal limitations of this study stems from the method used to identify HF cases, which relied exclusively on ambulatory data. This approach restricted our ability to apply the findings to a hospital-based context, making it impossible to accurately determine total annual expenditures and leaving us only with estimations of average annual costs per patient. Furthermore, the use of *DATASUS*, a secondary data source, introduced additional concerns regarding potential inaccuracies or incomplete information arising from errors made by healthcare providers during data entry. These considerations must be carefully weighed when interpreting the results and formulating conclusions about the study.

## CONCLUSIONS

The public healthcare expenditures in our sample differed between patients with diagnosed HF and those who are at risk of developing the condition, with potential HF accounting for the higher cost burden to the health system. Understanding the cost of this disease is important for improving resource allocation and implementing more effective measures for the population with HF.

## Additional material


Online Supplementary Document
