# Supplementary material for: Economic impact of heart failure in Brazil
Source: J Glob Health. 2026 May 15;16:04082. doi: 10.7189/jogh.16.04082 (PMC13178058; doi:10.7189/jogh.16.04082)

Supplement to: Volpi e Silva N, Vicentini D, Vitorasso R, Nogueira-Pileggi V, Bilevicius E, Batista V, Picoli R. Econonic Impact of heart failure in Brazil. J Glob Health. J Glob Health. 2026;16:04082.

Table S1. Percentage, over 5 years, in ambulatory and hospital system of diagnosed and potential HF Patients by procedure types.

		Ambulatorial system	Hospital system
**Procedures**	**Group**	**Total Cost (%)**	**Total Cost (%)**
**Diagnostic Procedures**	HF	24.90	0.27
	Potential HF	30.77	0.19
**Clinical Procedures**	HF	59.12	61.36
	Potential HF	57.92	29.20
**Surgical Procedures**	HF	4.98	35.56
	Potential HF	4.31	67.62
**Organ, Tissue, and Cell Transplants**	HF	2.05	2.81
	Potential HF	1.60	2.98
**Medications**	HF	5.38	-
	Potential HF	2.51	-
**Orthoses, Prostheses, and Special Materials**	HF	1.96	-
	Potential HF	1.63	-
**Complementary Health Care Actions**	HF	1.60	-
	Potential HF	1.25	-

Figure S1. Ranking of Medications in Ambulatory System by Number of patients and cost per patient for diagnosed and potential HF. (A) Total costs for diagnosed HF (B) Number of patients for diagnosed HF (C) Total costs for potential HF (D) Number of patients for potential HF.

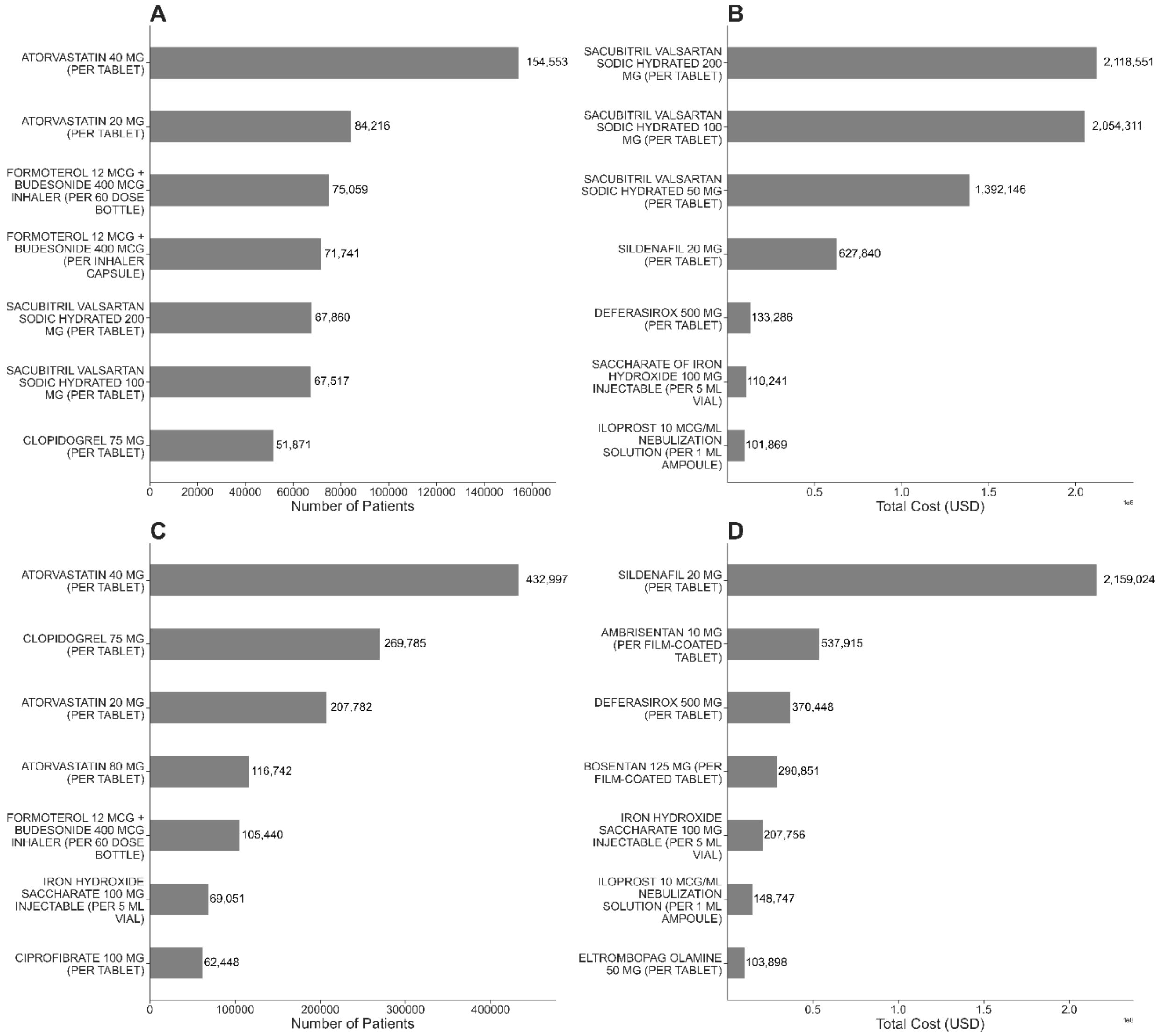

Figure S2. Ranking of Diagnostic Procedures in Ambulatory System by Total costs and Number of patients for diagnosed and potential HF. (A) Total costs for diagnosed HF (B) Number of patients for diagnosed HF (C) Total costs for potential HF (D) Number of patients for potential HF.

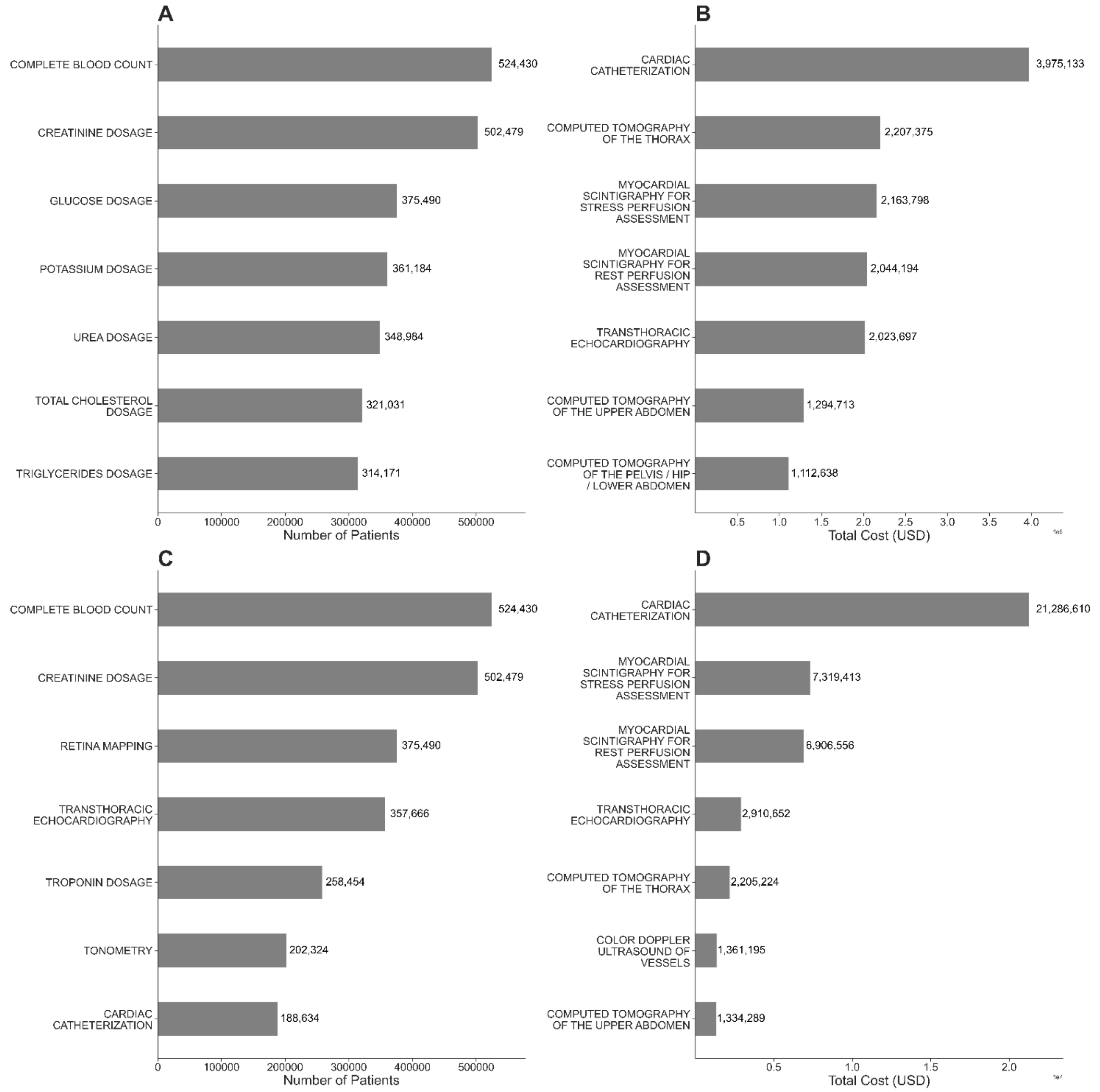

Figure S3. Ranking of Clinical Procedures in in Ambulatory System by Total costs and Number of patients for diagnosed and potential HF. (A) Total costs for diagnosed HF (B) Number of patients for diagnosed HF (C) Total costs for potential HF (D) Number of patients for potential HF.

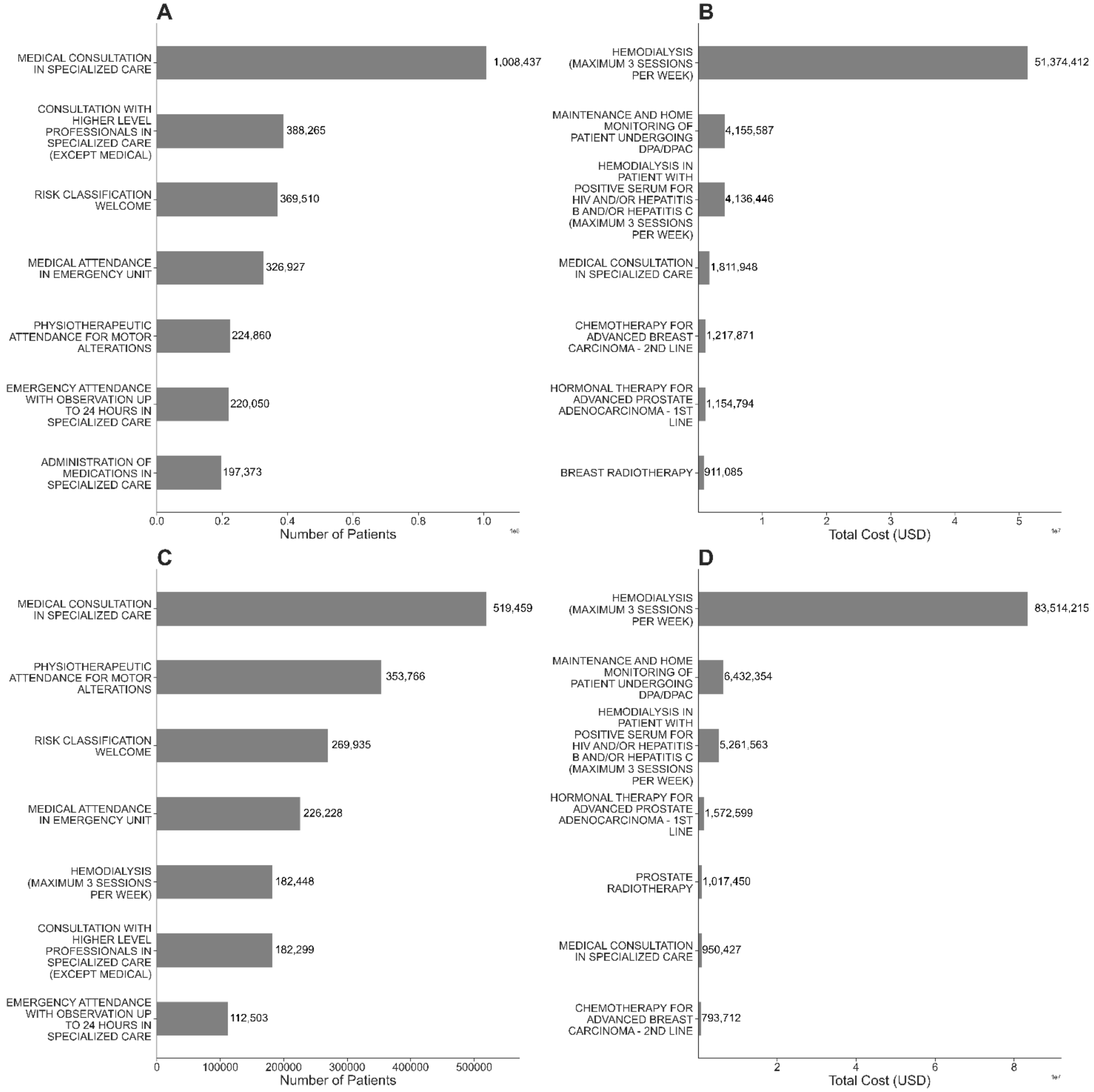

Figure S4. Ranking of Surgical Procedures in in Hospital System by Total costs and Number of patients for diagnosed and potential HF. (A) Total costs for diagnosed HF (B) Number of patients for diagnosed HF (C) Total costs for potential HF (D) Number of patients for potential HF.

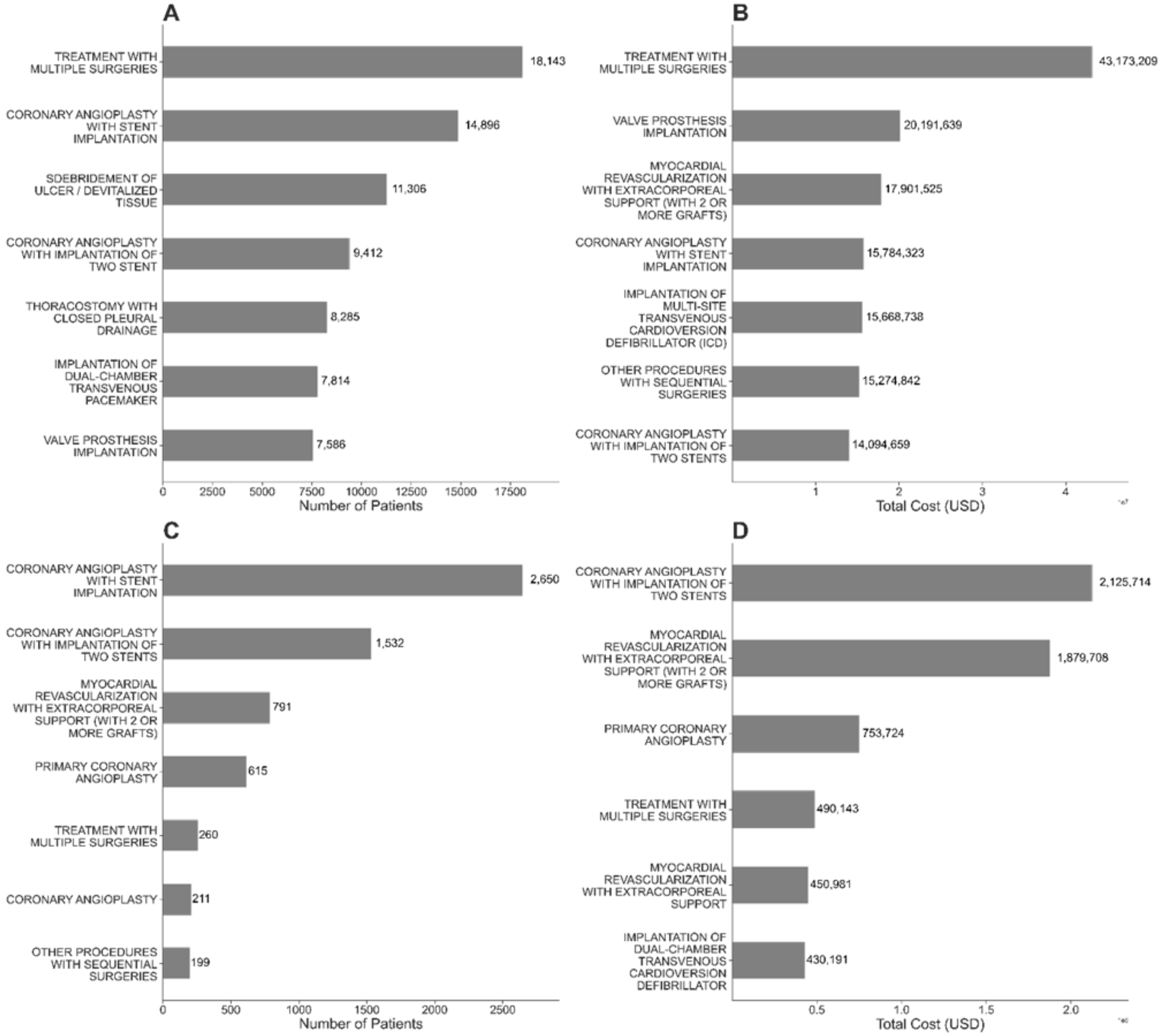

Figure S5. Ranking of Clinical Procedures in in Hospital System by Total costs and Number of patients for diagnosed and potential HF. **(A)** Total costs for diagnosed HF **(B)** Number of patients for diagnosed HF **(C)** Total costs for potential HF **(D)** Number of patients.

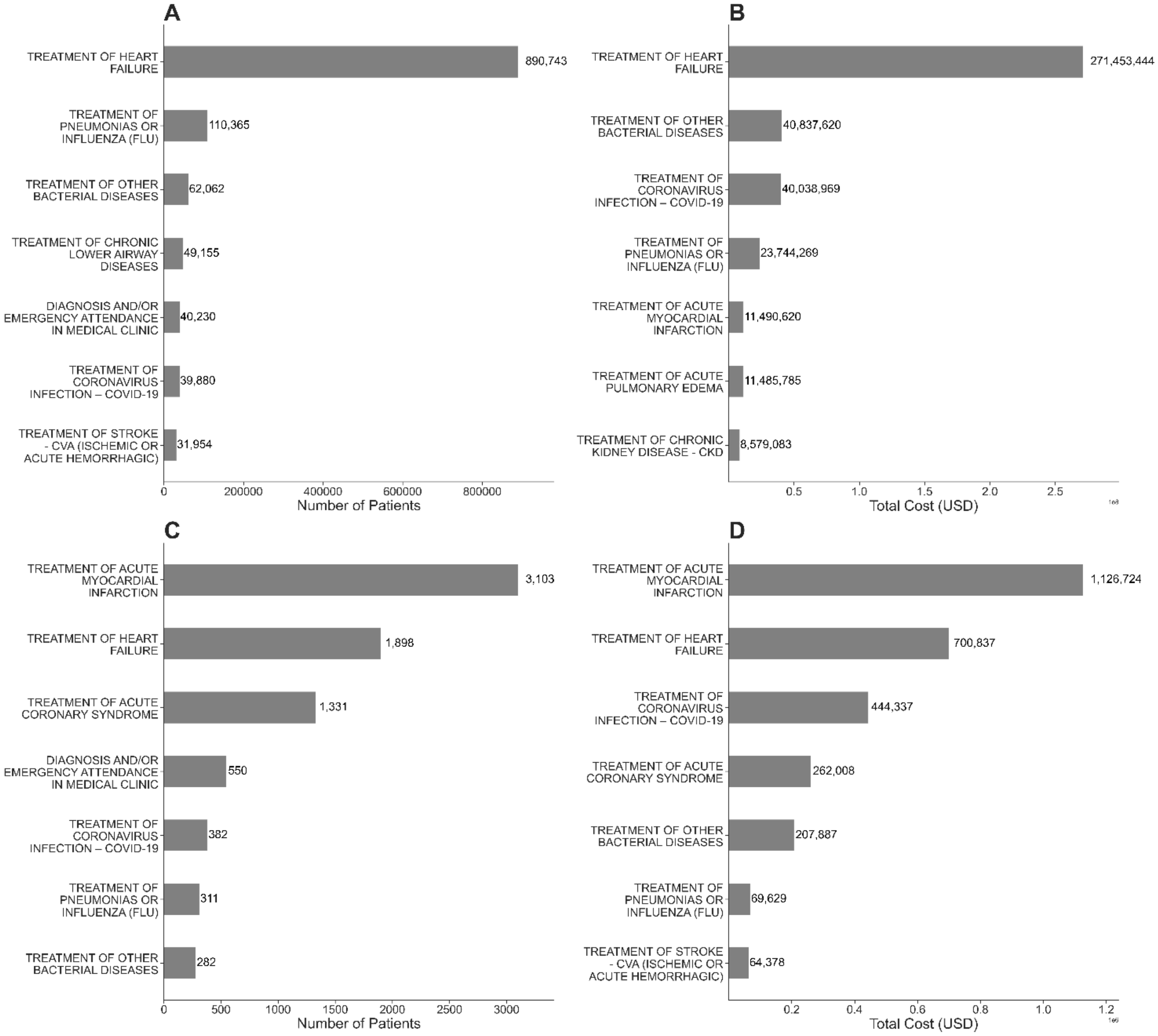

Figure S6. Choropleth map of cost per patient by region in the **hospital system**. **(A)** Diagnosed HF patients; **(B)** Potentials HF patients.

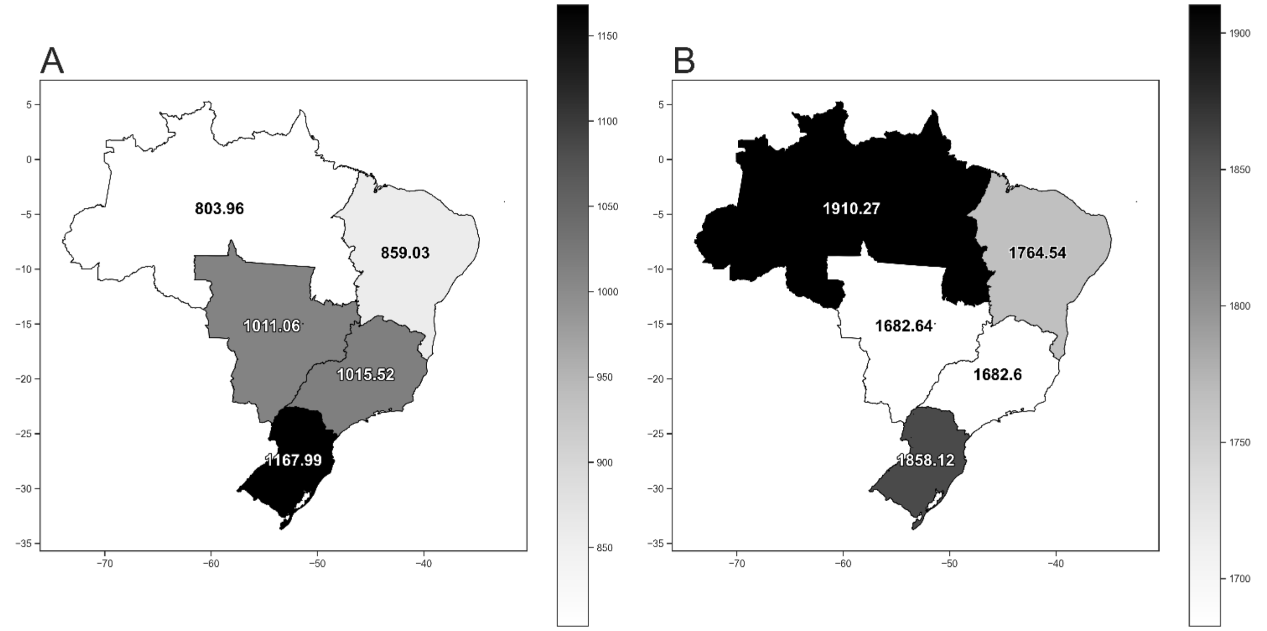

Figure S7. Choropleth map of cost per patient by region in the **ambulatory system**. **(A)** Diagnosed HF patients; **(B)** Potentials HF patients

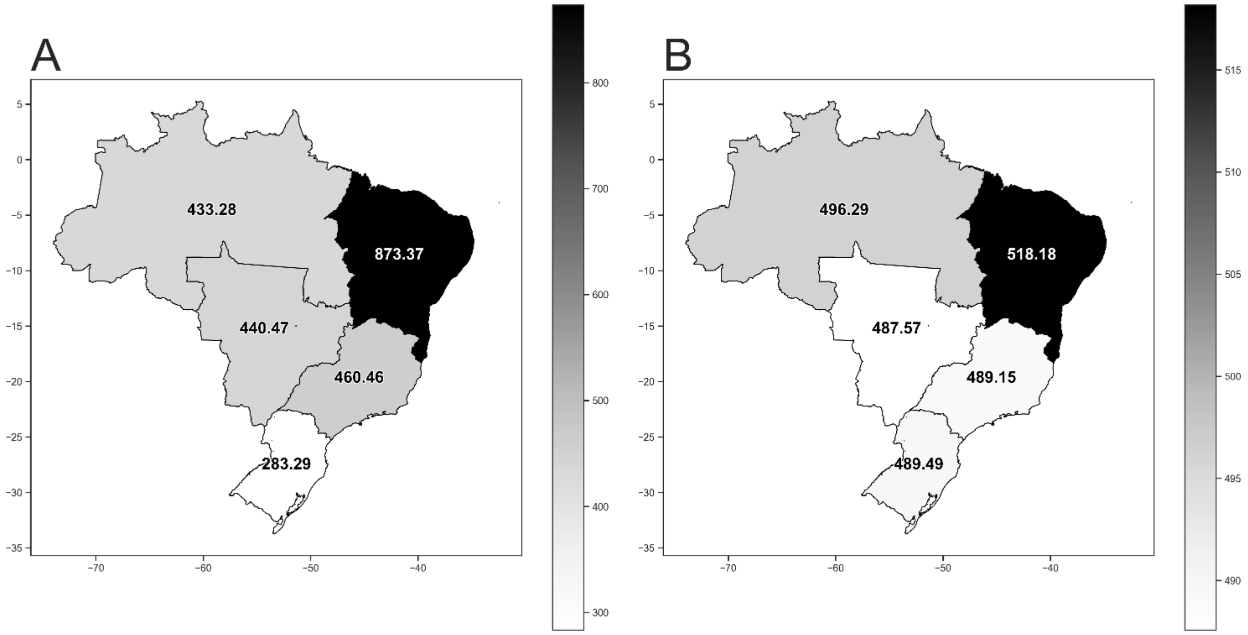

Supplement: Online Supplementary Document [file jogh-16-04082-s001.pdf]
